# Audiovestibular Findings in a Cohort of Patients with Chiari Malformation Type I and Dizziness

**DOI:** 10.3390/jcm12082767

**Published:** 2023-04-07

**Authors:** Hannah P. Famili, Christopher K. Zalewski, Alaaddin Ibrahimy, Jessica Mack, Fredric Cantor, John D. Heiss, Carmen C. Brewer

**Affiliations:** 1 Audiology Unit, NIDCD, National Institutes of Health, Bethesda, MD 20892, USA; 2 Department of Communication Science and Disorders, University of Pittsburgh, Pittsburgh, PA 15260, USA; 3 Surgical Neurology Branch, NINDS, National Institutes of Health, Bethesda, MD 20892, USA

**Keywords:** Chiari, Chiari Malformation Type I, vestibular, dizziness, balance, somatosensory, posturography, VEMP, SHA, rotational

## Abstract

Chiari Malformation Type I (CM1) is a neurological condition in which the cerebellar tonsils extend past the foramen magnum. While many studies have reported dizziness symptoms in patients with CM1, the prevalence of peripheral labyrinthine lesions is largely unknown. This study aimed to comprehensively describe the audiovestibular phenotype in a cohort of patients with CM1 expressly referred for dizziness. Twenty-four patients with CM1 and a complaint of dizziness/vertigo were evaluated. Hearing and auditory brainstem tract function were essentially normal. While vestibular abnormalities were most prevalent during rotational testing (33%), abnormal functional balance was the most common finding (40%). Patients with CM1 had a greater likelihood of exhibiting an abnormal sensory organization test (SOT) postural stability score for fixed platform conditions, and for the somatosensory analysis score. While no significant associations were identified between tonsillar ectopia extent and any vestibular/balance outcome measure, a significant negative association was identified between neck pain and the somatosensory sensory analysis score. Abnormal functional balance in the somatosensory domain was remarkable, with poorer scores associated with neck pain. An isolated peripheral vestibulopathy was present in only 8% of patients. Despite the low prevalence of vestibulopathy, vestibular/balance assessment is warranted to identify patients who may benefit from referral to specialized medical disciplines.

## 1. Introduction

Chiari Malformation type 1 (CM1) is an uncommon neurological condition with an estimated symptomatic prevalence of one in 1280 individuals (OMIM 118420). However, the exact prevalence of CM1 is challenging to determine because people with CM1 may have a subtle phenotype and be largely asymptomatic throughout their entire life [1]. A Chiari malformation (CM) occurs when the inferior part of the cerebellum, the cerebellar tonsils, protrudes through the foramen magnum and into the spinal canal. In 1891, Hans Chiari first described several forms of cerebellar abnormalities [2]. Six types of CM have since been described [3]. Four of these differ by the distance that the cerebellum and brainstem lie below the foramen magnum. Chiari Malformation Type I (CM1) is the mildest CM phenotype and is defined as at least 5 mm of cerebellar tonsillar ectopia below the foramen magnum [3]. The extent of ectopia is weakly associated with disease severity [4].

Type I is usually detected in early adulthood. It is often identified incidentally through an MRI to evaluate another condition such as headaches or injury [3]. Some CM1 patients have small posterior fossa volume, but others have tonsillar ectopia and a normal posterior fossa volume [5]. Inheritance patterns have suggested a genetic link to CM1 in families with reduced posterior fossa bone development [3]. Syringomyelia often accompanies CM1. Syringomyelia occurs in 30–70% of CM1 cases and is significantly related to the amount of tonsillar ectopia [5]. However, the severity of imbalance was independent of the presence or absence of syringomyelia [5].

Headache is the most common CM1 symptom [6,7,8]. Various other symptoms, including neck pain, extremity numbness and weakness, dizziness, ataxia, and fatigue are reported [7,9]. Studies have reported dizziness, imbalance, and vestibular symptoms in up to 70% of CM1 patients [5,7,9,10]. Aural fullness, tinnitus, and fluctuating hearing loss are less frequently reported [5]. However, the prevalence of peripheral labyrinthine lesions associated with CM-related dizziness and imbalance is unknown. Palamar and colleagues (2019) reported moderate imbalance and abnormal posturography-derived fall index scores in a cohort of CM1 patients [9]. They did not investigate the prevalence of peripheral vestibular lesions. Shaikh and Ghasia (2015) reported that gaze-evoked nystagmus (GEN) was the most prevalent ocular motor dysfunction, occurring in up to 30% of patients. Down-beating nystagmus, a form considered characteristic of craniovertebral junction disorders, was observed in only 4–6% of patients [6].

Most CM1 patients present initially with non-auditory complaints. However, aural fullness and tinnitus were reported in up to 81% of an otologic-referred CM1 patient cohort [5]. Within an otoneurologic setting, peripheral hearing loss occurred in approximately one-half of patients [5], but of unspecified type and degree. Cranial neuropathies occur in patients with CM1. Auditory brainstem responses are abnormal in as many as 75% of CM1 patients, suggesting the involvement of the eighth cranial nerve and its relays to the auditory cortex [11].

Previous studies have not fully detailed the auditory and vestibular manifestations of CM1. This study aimed to comprehensively describe the audiovestibular phenotype of CM1 in a cohort of patients referred for neurosurgical care of CM1 who reported dizziness. Understanding the type of auditory, functional balance, and vestibular manifestations as well as various associations with the severity of dizziness symptoms in patients with CM1 may help to provide further guidance for the management of patients with CM1-related dizziness.

## 2. Materials and Methods

### 2.1. Standard Protocol Approvals, Registrations, and Patient Consents

Studies were conducted at the National Institutes of Health (Bethesda, MD, USA) between 2012–2019 in compliance with the NIH Combined Neuroscience Institutional Review Board. Patients were enrolled in The Evaluation and Treatment of Neurosurgical Disorders (03-N-0164) or A Prospective Natural History Study of Syringomyelia or Associated Conditions (10-N-0143) clinical protocol. Written informed consent was obtained from all participants before enrollment.

### 2.2. Patients

Twenty-four patients with MRI-diagnosed CM1 and a complaint of dizziness, vertigo or imbalance were seen for comprehensive audiologic and vestibular testing. CM1 was radiologically defined as a cerebellar tonsillar descent 5 mm or greater below the McRae line [12]. The McRae line was drawn on the midsagittal section of an MRI scan between the anterior and posterior margins of the foramen magnum. The age distribution of the entire cohort did not deviate significantly from normal (*p* = 0.9822). Most patients were female (*n* = 21; 90%) (Table 1). This cohort’s 90% female predominance was higher than the 60% female CM1 predominance shown by Zhao and colleagues (2016). The age of the females (*Mdn* = 40.0, *SEM* 2.2) and males (*Mdn* = 49.0, *SEM* 9.4) did not differ significantly (*U* = 26.50, *z* = 0.44, *p* = 0.6858, *r* = 0.09).

### 2.3. Audiological Evaluation

The audiologic test battery included speech and pure-tone audiometry and middle ear function measures. Speech and pure-tone audiometry were conducted using GSI-61 (Grason-Stadler, Eden Prairie, MN, USA) clinical audiometers in a double-walled sound-treated room meeting American National Standards Institute (ANSI) criteria (ANSI S3.1-1999 (R2003)). Air and bone conduction pure-tone thresholds were obtained from 250–8000 Hz and 250–4000 Hz, respectively. Pure-tone threshold data were classified for hearing loss degree and type adapted from the European Working Group on the Genetics of Hearing Impairment (Table 2) [13]. The degree of hearing loss was based on a four-frequency pure-tone average (4F-PTA; 0.5/1/2/4 kHz). When hearing loss was present, the type of hearing loss was discerned by the air-bone gap, defined as the difference between the air and bone conduction three frequency pure-tone average (3F-PTA; 0.5/1/2 kHz). Criteria for determining the degree and type of hearing loss are presented in Table 2.

Middle ear function measures were conducted using a Grason-Stadler Tympstar (Eden Prairie, MN, USA) and consisted of 226 Hz tympanometry, acoustic stapedial reflex thresholds, and acoustic stapedial reflex decay. Acoustic stapedial reflexes, when tested, were interpreted as present, elevated, absent as expected, or absent but unexpected. Tympanometric criteria for determining middle ear function are presented in Table 2.

For a subset of 20 patients, a neurodiagnostic auditory brainstem response (ABR) was recorded using an 85 dB nHL click stimulus with an 8.1 click/sec repetition rate (Intelligent Hearing Systems; Miami, FL, USA). Criteria for normal ABR absolute and interpeak latencies are presented in Table 2.

### 2.4. Balance/Vestibular Evaluation

Balance function was assessed using computerized dynamic platform posturography via the NeuroCom SMART Equitest^®^ (previously Natus^®^). The comprehensive posturography test battery included measurement of Limits of Stability (LOS) and the Sensory Organization Test (SOT). SOT outcome measures included equilibrium postural stability scores across six conditions that rely on somatosensory (SOM), visual, (VIS) or vestibular (VEST) input. Sensory input is modified or removed for each of these three sensory modalities across all six conditions by which a composite and summary sensory analysis score is calculated for each modality (i.e., SOM, VIS, VEST) as well as a visual preference score (PREF). Test administration and determination of postural stability scores were performed accordingly to standard procedures [19,20]. Results from the SOT and LOS were used to report overall functional balance and to explore potential relationships to neck pain disability, headache severity, degree of cerebellar ectopia, syrinx location, and syrinx area.

Vestibular function was evaluated via rotational assessment and vestibular evoked myogenic potential testing. The vestibulo-ocular reflex (VOR) was measured by stimulating the horizontal semicircular canal during sinusoidal harmonic acceleration (SHA) at octave frequencies from 0.01–0.64 Hz using a calibrated Neuro Kinetics (Neuro Kinetics, Inc; NKI; Pittsburgh, PA, USA) Neuro-Otologic Test Center (NOTC). VOR suppression and vestibular–visual enhancement testing also occurred within the NOTC using 0.08–0.64 Hz, and at 0.08 and 0.64 Hz, respectively. All rotational stimuli were presented using NKI VEST™ software. Eye tracking was measured during ocular motor and rotational paradigms using NKI biocular Falcon™ infrared digital 250-Hz video goggles via I-Portal-VOG^®^ software. While video head impulse testing (vHIT) has increased the stimulus range of vestibular responses above 1Hz, this test was deferred secondary to a concern for exacerbating neck pain and subsequent associated symptoms in this cohort.

Cervical and ocular vestibular evoked myogenic potentials (cVEMPs and oVEMPs) were elicited via an air-conducted 500 Hz tone burst stimulus (Blackman gating, 2 ms rise/fall time, 0 ms plateau) presented monaurally via insert earphones at 100–107 dB nHL and a repetition rate of 5.1 bursts per second (Intelligent Hearing Systems; Miami, FL, USA). Myogenic activity during the cervical VEMP was recorded from surface electrodes placed on the ipsilateral sternocleidomastoid (reference), the sternum (active), and the forehead (ground). The ipsilateral sternocleidomastoid electromyogenic activity was continuously monitored during testing and accepted when electromyogenic responses were between 50–100 μV. Myogenic activity during the ocular VEMP was recorded from surface electrodes from the contralateral inferior oblique using the belly-tendon montage [21]. The cervical and ocular VEMPs were interpreted based on the presence or absence of the bi-phasic P1-N1 peak response and interaural symmetry ratio of the P1-N1 amplitude.

Normal reference ranges and classification criteria for all balance and vestibular assessments are presented in Table 2. For SHA and c/oVEMP groupwise analyses, data were compared against NIH control data. While the mean age of the Chiari cohort (*Mdn* = 40.0, *SEM* 2.2) was significantly older than the NIH control cohort (*Mdn* = 24.0, *SEM* 1.2) *U* = 147.5, *z* = 1.24, *p* ≤ 0.0001, *r* = 0.14, age has not shown a significant relationship to vestibular outcome measures except for a negative relationship with VEMP amplitude [22]. To avoid older-age-related postural stability effects, posturography groupwise and contingency analyses were compared against NIH controls between the ages of 20 and 59. While the Chiari cohort (*Mdn* = 40.0, *SEM* 2.2) was significantly older than the NIH posturography control cohort (*Mdn* = 32.0, *SEM* 2.2) *U* = 220.5, *z* = 0.2582, *p* ≤ 0.0498, *r* = 0.04, the effect size was small.

### 2.5. Neurological Data

Scores on the Neck Pain Disability Index (NDI) questionnaire were used as an indicator of the severity of neck pain and headache and to record neck pain disability [18]. A history of previous Chiari decompression surgery, the extent (mm) of cerebellar ectopia, and presence of an associated syrinx were obtained from the neurology and physical history (J.C., M.D.).

### 2.6. Statistical Analysis

Descriptive and inferential statistics were computed using Microsoft Excel (v16.6) and GraphPad Prism version 9.3.1 for Mac, GraphPad Software, San Diego, CA, USA. Descriptive statistics were used to compute prevalence or normal and abnormal findings. Comparison of CM1 data against NIH normative reference ranges for rotational, cVEMP, and posturography data, nonparametric inferential analyses were performed using Mann–Whitney U test, Kruskal–Wallis *H* one-way analysis of variance with a Tukey’s test for multiple comparisons, and Fisher’s Exact test. Spearman’s rank correlations were performed to investigate associations between vestibular outcome measures and neurological data. The level of significance was set at α < 0.05.

## 3. Results

### 3.1. Auditory Results

**Figure 1:**
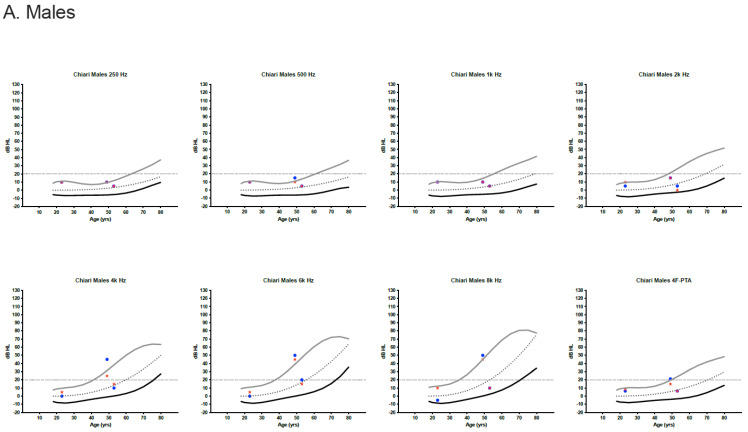

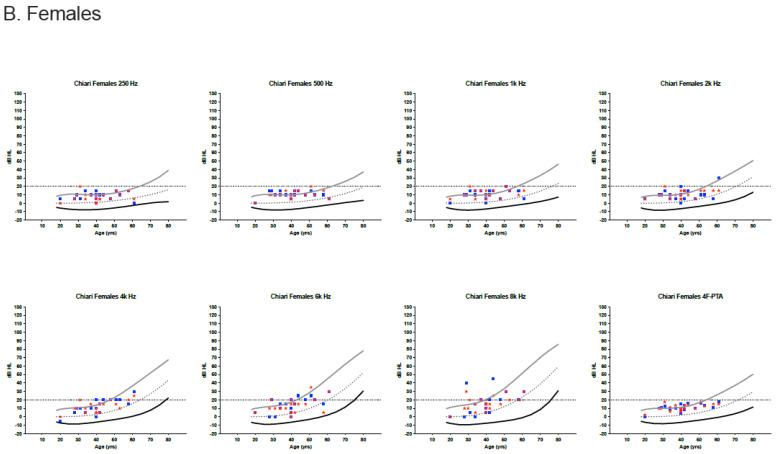
Audiometric thresholds in dB HL for males (**A**) and females (**B**) plotted by age [23]. Curved lines represent the International Organization for Standardization (ISO) normative age-distributions for the 5th percentile (black line), the median (dotted line) and the 95th percentile (grey line) for each sex and frequency, including the four-frequency pure-tone average (4F-PTA). Horizontal dotted line at 20 dB HL identifies the upper limit for normal clinical hearing determination. Red stars represent the right ear and blue circles/boxes represent the left ear.

Behavioral audiometric evaluations were completed on 23 of 24 patients (96%). Word recognition scores were within normal limits (≥88%) for all patients. Clinically normal hearing using the 4F-PTA was documented in 22 of 23 patients (95.7%). The remaining patient had a mildly elevated left ear 4F-PTA (21.25 dB HL) and a history of noise exposure. High-frequency hearing loss was present bilaterally for four individuals and unilaterally for a single individual. Most pure-tone thresholds were within the 95th percentile for the International Organization for Standardization (ISO) normative age distributions (Figure 1) [23].

For those pure-tone thresholds exceeding the 95th percentile limits, responses often remained within the clinically normal hearing limits.

Tympanometry was normal bilaterally in all 30 patients. Acoustic stapedial reflexes were present at expected levels with no evidence of abnormal acoustic reflex decay for all individuals tested.

Auditory Brainstem Responses (ABRs) were recorded in 20 of the 24 patients. All ABR data were normal except for isolated prolongations in the absolute latency of wave III in a right ear (4.18 ms) and left ear (4.16 ms) of two individuals, one resulting in a delay of 0.04 ms in the right ear wave I–III interpeak latency. In both cases, the wave V absolute latency and the wave I–V interpeak latency were within normal limits, limiting the auditory neurodiagnostic relevance of the wave III delays in both ears. A summary of the audiometric results for each patient is presented in Table 3.

### 3.2. Vestibular Results

Most patients (*n* = 15; 62.5%) completed over half of the vestibular test battery (five to eight tests). Four of 24 patients (16.7%) patients completed the entire balance and vestibular test battery (nine tests), while five patients (20.8%) received less than half the test battery (less than five tests). Test sessions were incomplete for a variety of reasons including time constraints, scheduling conflicts, and patient intolerance of rotational tests (e.g., visual-vestibular enhancement, high-velocity step testing), and concern for excessive acoustic exposure. While most (19/24, 79.2%) patients received cVEMP testing, fewer received oVEMP testing (*n* = 10) secondary to a concern for excessive acoustic exposure [24]. Overall, functional balance deficits evidenced by SOT abnormalities were most prevalent (8/23; 39.1%), followed by rotational abnormalities (8/24; 33.3%) and cVEMP abnormalities (2/19, 10.5%). A comprehensive summary of each patient’s and the cohort’s vestibular results is shown in Table 3 and Table 4, respectively.

### 3.3. Platform Posturography

Using published normative ranges [16] (NeuroCom SMART Equitest^®^ (Natus^®^)), posturography identified functional balance deficits in over one-third of patients (9/23; 39.1%). Individuals exhibited difficulty maintaining normal postural stability in vestibular dependent environments (7/23; 30%), somatosensory dependent environments (6/23; 26%), and vision dependent environments (3/23; 13%).

Patients with reduced sensory analysis scores had no clear postural deficit pattern across all three sensory modalities. Two of the nine patients had isolated reduced somatosensory modality scores, two of nine isolated reduced vestibular modality scores, but none had an isolated reduced visual modality sensory analysis score. Three of the remaining five patients had poor postural sensory analysis scores across two sensory environments. Two of the five had reduced postural sensory analysis scores across all modalities (somatosensory, vestibular, and visual). Only one of the seven patients with a low vestibular sensory analysis score had corroborating evidence supporting a peripheral vestibulopathy (reduced VOR gain with concomitant increased low-frequency VOR phase leads). Four of the remaining six patients with reduced vestibular sensory scores had concomitant evidence supporting a mixed or unknown site-of-lesion and two of a central lesion. Of the two individuals within the entire cohort with evidence of an isolated peripheral lesion, one had an isolated low vestibular sensory score (previously noted), and the other an isolated low somatosensory score. A summary of the posturography data for each patient is presented in Table 3.

### 3.4. Rotational Testing

Rotational testing was performed on all 24 individuals. Of the rotational tests, SHA was the test identifying the most (*n* = 8) abnormal patients, of which five had an abnormality impacting VOR phase lead. Four of these exhibited an increase, and the other patient showed a decrease in the VOR phase lead. Two of the four patients with an increase in the VOR phase lead also had a reduction in low-frequency VOR gain, consistent with a peripheral vestibulopathy, and the two remaining individuals had concomitant normal VOR gain suggestive of a compensated peripheral vestibulopathy. The remaining patient with an abnormal decrease in VOR phase lead exhibited concomitant VOR gain asymmetry, consistent with a central lesion.

In addition to the previously noted two patients with a documented decrease in VOR gain, there was an additional patient with an increase in low-frequency VOR gain and failure of angular VOR suppression, which, collectively, are consistent with a central lesion within the cerebellum. This patient was the only one with abnormal angular VOR suppression. Finally, two of 13 patients (15.4%) had increased visual-vestibular enhancement rotational gain. SHA testing showed that both patients also had associated abnormal VOR gain asymmetry. High-velocity step testing provided no evidence of any labyrinthine asymmetries. Shortened low-velocity VOR time constants were associated with VOR phase abnormalities in two patients (Table 3).

### 3.5. VEMP Testing

Two of 19 patients (10.5%) undergoing cVEMP testing had an abnormal cVEMP response. One of these had an abnormal prolongation in the P1 latency, and the other had *increased* right ear P1-N1 amplitude. Neither patient had any concomitant abnormal vestibular findings. However, they had abnormal SOT sensory scores, an abnormal isolated somatosensory score in one and an abnormal visual/vestibular score in the other. No patients had a reduced P1-N1 cVEMP amplitude or abnormal P1-N1 cVEMP amplitude ratio. The oVEMP was normal in the ten patients undergoing this testing.

### 3.6. Site-of-Lesion Analysis

Vestibular test results were evaluated to assign a suspected site-of-lesion according to operational definitions described in Table 2. The site-of-lesion was central in 16.7%, peripheral in 8.3%, unknown or non-localizing in 29.2%, and without lesion confirmation based on normal vestibular function testing in 45.8% of patients. The two individuals with peripheral vestibular dysfunction had low VOR gain, increased low-frequency VOR phase lead, and abnormal VOR time constants on rotational testing. Interestingly, both patients had normal cVEMP and oVEMP findings (Table 3).

### 3.7. Groupwise Analysis

**Figure 2:**
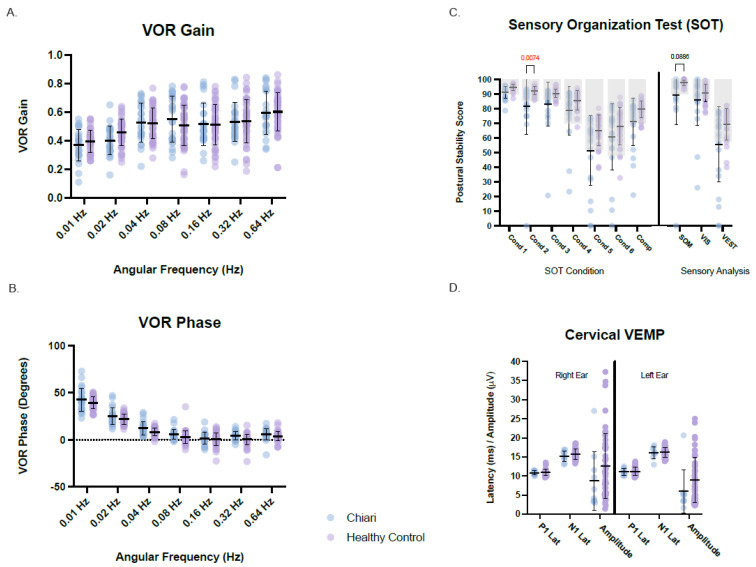
Vestibular and balance results for rotational VOR gain (**A**), rotational VOR phase (**B**), sensor organization test (**C**), and cervical VEMP (**D**). Chiari cohort data is plotted in light blue and the NIH healthy control data is plotted in light purple. Means ±1 standard deviation are plotted for each. Manufacturer normative ranges are indicated by light grey boxes for the sensory organization test (**C**). When present, *p*-values are identified by brackets. Hz = Hertz, Lat = latency, Cond = Condition, Comp = composite score, SOM = somatosensory, VIS = visual, VEST = vestibular, PREF = visual preference.

Individual descriptive analyses identified various discrete abnormalities among patients across most assessments. On the other hand, groupwise comparisons against available normative reference ranges obtained on healthy volunteers at the NIH (normal controls) identified only a few significant differences (Figure 2). The Chiari group’s data was compared to normal controls for the Sensory Organization Test (SOT), SHA measures of VOR gain, phase, and symmetry, and ocular and cervical VEMP measures of absolute P1-N1 amplitude, latency, and amplitude symmetry ratio for each ear. There were no significant groupwise differences between the Chiari group and normal control group for any vestibular outcome measure, with a few exceptions. The Chiari group had a significantly lower score for SOT condition 2 than the healthy control group (*H*(14) = 277.3, *p* = 0.0087, *z* = 0.348, *r* = 0.05). While not significant, there was a consequent lower somatosensory SOT sensory analysis score for the Chiari group when compared to NIH controls (*H*(8) = 141.0, *p* = 0.0886, *z* = 0.1325, *r* = 0.02) (Figure 2). Moreover, Fisher’s exact test shows a greater likelihood for an individual with a Chiari malformation to have an abnormal SOT postural stability score for condition 1 (*p* = 0.0339, *OR* 0.09), condition 2 (*p* ≤ 0.0001, *OR* 0.02), condition 3 (*p* = 0.0185, *OR* 0.17), and for the somatosensory analysis score (*p* = 0.0014, *OR* 0.03) when compared to the NIH Control for 20–59-year-old individuals.

The Chiari group had a lower mean cVEMP amplitude for both the right and left ears compared to the control group. While this difference did not reach statistical significance, the observed difference could be attributed to the mean age of the Chiari cohort exceeding that of the NIH controls [22]. Summary groupwise comparisons between Chiari and normal controls for posturography, rotational tests, and cVEMP responses are presented in Figure 2.

### 3.8. Neurological Symptom Analyses

**Figure 3:**
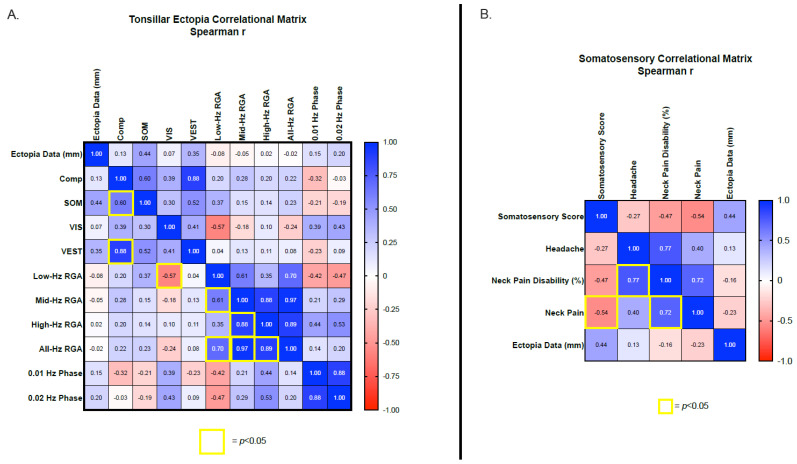
Correlational matrix between tonsillar ectopia data and vestibular/balance outcome measures (**A**) and between the SOT somatosensory analysis score and neurological symptoms (**B**). Significant *p*-values (<0.05) are highlighted by yellow boxes and effect size (*r*) is provided for each association. Color shading relative to effect size from −1.0–1.0. Low-Hz. RGA = low-frequency rotational average VOR gain for 0.01, 0.02, and 0.04 Hz. Mid-Hz RGA = mid-frequency rotational average VOR gain for 0.04, 0.08, and 0.16 Hz. High-Hz RGA = high-frequency rotational average VOR gain for 0.16, 0.32, and 0.64 Hz. All-Hz RGA = rotational average VOR gain for 0.01–064 Hz. Comp = Sensory organization test composite score, SOM = somatosensory sensory analysis score, VIS = visual sensory analysis score, VEST = vestibular sensory analysis score.

Potential associations between the hallmark finding of CM1, tonsillar ectopia, and all the vestibular outcome measures were investigated using multiple regression correlation matrices. No significant associations were identified between the amount of tonsillar ectopia and any vestibular or postural stability outcome measures (Figure 3A). Similar analyses investigated associations between the NDI severity scores for headache, neck pain, neck pain disability with SOT sensory analysis measures (i.e., somatosensory, vestibular and visual). An isolated significant negative association was identified between the severity of reported neck pain and the SOT somatosensory sensory analysis score (*p* = 0.0272, *r* = −0.5397), with greater neck pain associated with a poorer somatosensory sensory analysis score (Figure 3B). Although neck pain and the neck pain disability score were strongly associated (*p* = 0.002, *r* = 0.7230), the neck pain disability score and the SOT somatosensory sensory analysis score were not associated (*p* = 0.061, *r* = −0.4650). Neck pain disability was strongly associated with headache severity (*p* = 0.0004, *r* = 0.7730). However, headache severity was not associated with neck pain or somatosensory, visual, and vestibular SOT sensory analysis scores.

Various contingency analyses explored potential associations between the presence or absence of a syrinx and vestibular and balance outcomes. The presence or absence of a syrinx was not associated with a normal or abnormal SOT sensory score (somatosensory, visual, or vestibular) or SOT composite score. Additionally, the presence or absence of a syrinx was not associated with a normal or abnormal cervical VEMP. However, a cervical syrinx was present in the two cases where an abnormal cVEMP was present. Finally, SOT sensory analysis scores were compared between the cervical syrinx CM1 group and the non-syrinx CM1 group. Postural stability in all postural sensory domains of the SOT was not significantly different between individuals with and without a syrinx. Moreover, neck pain severity, headache severity, and neck pain disability scores also did not significantly differ between the CM1 groups with and without a cervical syrinx.

## 4. Discussion

### 4.1. Audiovestibular Phenotype

Dizziness, imbalance, and vestibular symptoms have been reported in up to 70% of CM1 patients [7]. Medical articles about peripheral labyrinthine lesions associated with CM-related dizziness and imbalance are confined mainly to individual case reports. Guerra Jimenez et al. (2015) described two cases of recurrent vertigo with peripheral features. Weber and Cass (1993) documented peripheral vestibular dysfunction in CM1 patients by reduced unilateral caloric responses [10,25]. Weber and Cass (1993) also reported central vestibular nystagmus in three patients [25]. Only 2 of 24 (8%) patients with CM1 in our study had distinct peripheral vestibular lesions by vestibular testing. This prevalence is particularly low given that all patients referred for vestibular and balance testing in this cohort had dizziness or vertigo symptoms. Despite the high prevalence of audiovestibular symptoms in patients diagnosed with CM1 [6], our results indicate that these symptoms are usually unrelated to peripheral vestibular pathology. Instead, our vestibular and balance function testing data support that the dizziness phenotype more often arises from central vestibular pathway pathology.

This study failed to show an overt auditory phenotype. However, patients with CM1 in this cohort were referred for vestibular and balance testing secondary to dizziness and not hearing dysfunction, which likely limits the generalization of these data beyond this referral group of patients with CM1 specifically referred for dizziness. Nevertheless, the essential absence of any significant peripheral hearing loss is noteworthy as some patients had migraine and headache co-morbidities, and subjective hearing loss is more likely in patients with migraine [26]. The absence of retrocochlear abnormalities on ABR suggests that the auditory brainstem tracts are unaffected in patients with CM1 who report dizziness or vertigo.

Unlike patient groups with diseases and syndromes (e.g., Neurofibromatosis Type II, Enlarged Vestibular Aqueduct) that produce marked vestibular and balance abnormalities compared to healthy individuals, our patient group had vestibular outcome measure results that did not differ from healthy controls. However, the balance data analysis identified significantly lower somatosensory postural functional stability in our cohort when compared to healthy controls. We also found a significant association between reduced somatosensory postural stability scores and the report of worsening neck pain, a condition often referred to as cervical dizziness [27]. These data suggest increased balance dysfunction in patients with CM1 is associated with Chiari-related neck pain. Previous reports have corroborated similar conclusions documenting increased balance dysfunction in patients with CM1 [7,9], however, this is the first study showing significant associations between reduced somatosensory postural stability scores and worsening neck pain using SOT. While increased sway during quiet stance with concomitant disruption of blood flow to the ankles has been previously reported and may serve as a model for the observed increased somatosensory sway in patients with Chiari and increased neck pain [28], more research is needed to further elucidate this hypothesis. Although there is no demonstrable effect of a cervical syrinx on balance symptoms, as abnormal cVEMPs were not associated with the presence or absence of a cervical syrinx, this finding’s generalizability may be limited by its small sample size and low statistical power.

No single test was a “gold standard” test for assessing dizziness in patients with CM1. Instead, abnormal vestibular results were found in various assessments. Abnormalities in vestibular function were most prevalent during posturography and rotational testing, particularly in identifying test patterns consistent with central dysfunction, such as VOR Suppression, visual-vestibular enhancement, and VOR phase abnormalities during SHA. While abnormal findings support a primary reduction in postural stability involving somatosensory and vestibular function during posturography, one must recognize that posturography is a functional assessment of balance that does not definitively localize the anatomical site-of-lesion.

The cVEMP may appear more sensitive than oVEMP because no patient with CM1 had an abnormal oVEMP, and two of 19 (10.5%) patients had an abnormal cVEMP. This conclusion, however, should be interpreted with caution given the small sample size and non-significant associations between VEMP and the presence or absence of a cervical syrinx. A larger cohort of patients with CM1 would provide greater statistical power to explore whether the presence or absence of cervical syringes was associated with an abnormal cVEMP.

### 4.2. Neurological Vestibular Relationships

Recent findings suggest that the extent, in mm, of tonsillar ectopia is weakly associated with the severity of CM1 disease symptoms [4]. In our study, we found no significant association between the extent of tonsillar ectopia and vestibular or balance outcomes. Although our small cohort size may limit the generalization of this finding, one could postulate that the requisite inclusion criterion of dizziness would have served to only strengthen an alternate *a priori* hypothesis for the existence of such a relationship. Additional investigation into a phenotype-ectopia relationship is warranted, including a larger sample that considers the size, location, and volume of any concomitant syrinxes [29].

It remains uncertain if balance function or dizziness symptoms improve after Chiari decompression surgery. Remediation of dizziness complaints have been reported following surgical decompression [7,30]. However, improvement may be transient with dizziness returning in some patients [31,32]. Five individuals within our cohort were seen post decompression surgery; all had complaints of dizziness at that time. Regrettably, only two patients were seen pre-, and post-surgical decompression, and such a small sample is not amenable to statistical analysis. Future investigation before and after surgery is warranted to determine if surgical intervention impacts audiovestibular objective measures.

## 5. Conclusions

In a cohort of patients with CM1 expressly referred for dizziness, approximately half the patients had normal vestibular and balance function. An isolated peripheral vestibulopathy was present in only 8% of patients. However, if those individuals with a non-localizing site-of-lesions were considered, the prevalence of a possible peripheral contribution could extend to as much as 38%. Evidence for central contribution to the dizziness in this cohort was present in almost half of the cohort when considering those with a non-localizing site-of-lesion. Hearing sensitivity and auditory brainstem tract function was essentially normal.

A heterogeneous profile of abnormal test findings was present across all vestibular assessments. No “gold-standard” test identified or contributed to an apparent vestibular phenotype. Abnormal functional balance in the somatosensory domain was remarkable, with poorer somatosensory scores significantly associated with the degree of neck pain.

Overall, these data suggest that dizziness usually originated from the CNS in our group of patients with CM1. Abnormal functional balance in the somatosensory domain was remarkable, with poorer scores associated with neck pain. Although the low prevalence of an evident peripheral vestibular lesion, vestibular and balance assessment is still warranted for appropriate referrals to specialized medical and rehabilitative disciplines. Continued research is needed to investigate the effects of surgical intervention and the relationships between cerebellar dysfunction and patient symptoms of dizziness, neck pain, and headache often comorbid with Chiari Malformation.

## Figures and Tables

**Table 1:** Patient Demographics.

	Sample Size (n)	Age Range (yrs)	Mean (yrs)	SD (yrs)	Median (yrs)	IQR (yrs)
Male	3	23–53	41.67	16.29	49	23–53
Female	21	20–61	40.76	9.93	40	34–46
**Total**	**24**	**20–61**	**40.88**	**10.44**	**40**	**34–48.75**

**Table 2:** Classifications and criteria for hearing and vestibular outcome measures.

Outcome Measure		Criteria
**HEARING**		
**Degree of HL ^a^**		
None		≤20 dB HL
Mild		>20 and ≤40 dB HL
Moderate		>40 and ≤70 dB HL
Severe		>70 and ≤95 dB HL
Profound		>95 dB HL
**Type of HL ^b^**		
Conductive		AC >15 dB HL; BC ≤ 15 dB HL; ABG > 10 dB
Mixed		BC >15 dB HL; ABG > 10 dB
Sensorineural		AC >15 dB HL; ABG ≤ 10 dB
**Tympanometry ^c^** [14]		
Normal (Type A)		0.3–1.4 mmho > −100 daPa
Immobile (Flat) (Type B)		No mobility, no peak
Negative Pressure (Type C)		<−100 daPa
Hypermobility (Type Ad)		>1.4 cc, >−100 daPa
Hypomobility (Type As)		<0.3 cc, >−100 daPa
**Auditory Brainstem Response ^d^** [15]		Abnormal when
Absolute latency I, III, V		>1.79 ms, 4.08 ms, 6.08 ms, respectively
Interpeak latency I–III, III–V, I–V		>2.60 ms, 2.26 ms, 4.49 ms, respectively
Interaural wave V latency difference		>0.40 ms
**VESTIBULAR**		
**Posturography**		
SOT		Abnormal when below 95th percentile limits ^e,h^ [16]
**Rotational Assessment**		
Gain	0.01–0.64 Hz	Abnormal value isolated to 0.01 Hz (gain and phase) or involving two consecutive octave frequencies from 0.01–0.64Hz ^f^ [17]
Phase		
Symmetry		
60° Velocity Step		10 s > Abnormal Time-Constant > 32 s with concurrent interpretation of estimated TC from VOR Phase.
240° Velocity Step		Abnormal peak eye velocity symmetry ≥20%
Suppression (0.08–0.64 Hz)		Abnormal value involving a single frequency
Visual-Vestibular Enhancement (0.08 Hz and 0.64 Hz)		Abnormal value involving a single frequency
**Cervical VEMP**		
P1-N1 Amplitude Ratio		Abnormal > 35%
**Ocular VEMP**		
P1-N1 Amplitude Ratio		Abnormal > 40%
**NEUROLOGICAL SYMPTOMS**		
Headache Rating		Headache Pain Score (Section 5) ^g^ [18]
Neck Pain Disability Rating		Neck Pain Disability Questionnaire Percentage ^g^ [18]
Neck Pain Intensity Rating		Neck Pain Score (Section 1) ^g^ [18]
Surgical History		Surgical history prior to assessment (Y/N)
**SITE OF LESION Interpretation**		
Normal		All tests WNL
Peripheral		Reduced (or unexplained absent) VEMP amplitude; Reduced VOR SHA Gain which may include an increase in VOR Phase with or without a VOR Asymmetry and an abnormal low-velocity step
Central		Increased VEMP amplitude; Increased VOR SHA Gain; Abnormal Enhancement and/or VOR Suppression; Decreased VOR Phase with or without VOR Asymmetry
Unknown (Non-Localizing)		Isolated Increased VOR Phase with or without VOR Asymmetry and/or low-velocity step abnormality; Isolated VEMP Latency abnormality; Isolated VOR Asymmetry
Mixed		Evidence meeting components of both peripheral and central

AC, air conduction; BC, bone conduction; ABG, air-bone gap; PTA, pure-tone average; freq, frequency; three-frequency (0.5, 1, 2 kHz); four-frequency (0.5, 1, 2, 4 kHz); ^a^ based on 4F-PTA by AC; ^b^ based on 3F-PTA for AC and BC; ^c^ Margolis and Heller, 1987, respective Jerger type provided in parentheses; ^d^ Normative values are ±2.5 SD from Schwartz et al. (1989) [15]: ages 19–36 years; 80 dB nHL broadband click stimulus; rarefaction and condensation polarities combined; ER-3A transducer; ^e^ NeuroCom SMART Equitest (Natus^®^, Pleasanton, CA, USA), Borah et al., 2007; ^f^ Zalewski, 2018; ^g^ Vernon et al., 1992; ^h^ NIH Normative Data, adapted dataset confined to 20–59 yr age range, *n* = 31.

**Table 3:** Summary of Audiovestibular Results.

CM #	Age	Sex	cVEMP	oVEMP	VOR Gain	VOR Phase	VOR Sym	VOR Supp	VOR Enhance	60° Velocity Step Data	240° Velocity Step Data	Site-of-Lesion	SOM	VIS	VEST	SOT-Comp	A-P LOS	PTA		ABR	SPCH
																		LE	RE		
**1**	31	F	DNT	DNT	WNL	WNL	WNL	DNT	DNT	DNT	DNT	Unknown	85	26	0	21	DNT	12.50	17.50	WNL	WNL
**2**	48	F	DNT	DNT	WNL	WNL	WNL	DNT	DNT	DNT	WNL	Normal	96	84	69	79	DNT	10.00	10.00	WNL	WNL
**3**	49	M	WNL	WNL	WNL	WNL	WNL	WNL	DNT	WNL	WNL	Normal	98	95	78	86	DNT	21.25	15.00	WNL	WNL
**5**	40	F	ABNL-R Lat	DNT	WNL	WNL	WNL	DNT	DNT	WNL	CND	Unknown	81	98	61	81	DNT	3.75	6.25	DNT	WNL
**6**	40	F	DNT	DNT	WNL	ABNL-Dec	ABNL	DNT	DNT	WNL	WNL	Central	DNT	DNT	DNT	DNT	DNT	15.00	7.50	WNL	WNL
**7**	42	F	ABNL-R Amp (High)	DNT	WNL	WNL	WNL	DNT	DNT	WNL	WNL	Unknown	95	47	13	41	DNT	8.75	10.00	WNL	WNL
**8**	28	F	WNL	WNL	WNL	WNL	WNL	WNL	WNL	WNL	WNL	Normal	90	89	78	82	8.4	10.00	10.00	WNL	WNL
**12**	53	F	WNL	WNL	WNL	WNL	ABNL	WNL	ABNL-High	WNL	WNL	Central	87	85	52	71	10.4	13.75	12.50	WNL	WNL
**13**	40	F	WNL	WNL	WNL	WNL	WNL	WNL	WNL	WNL	WNL	Normal	98	89	69	81	DNT	8.75	7.50	WNL	WNL
**14**	51	F	WNL	WNL	ABNL-Low	ABNL-Inc	WNL	WNL	WNL	ABNL-Low (3/4)	WNL	Peripheral	87	95	63	73	8.9	16.25	17.50	DNT	WNL
**15**	20	F	WNL	WNL	WNL	WNL	WNL	WNL	WNL	ND	ND	Normal	96	95	72	82	10.7	0.00	2.50	WNL	WNL
**17**	42	F	WNL	DNT	WNL	WNL	WNL	WNL	DNT	WNL	ND	Normal	98	100	75	74	8.7	13.75	13.75	ABNL	WNL
**19**	40	F	WNL	DNT	WNL	WNL	ABNL	WNL	ABNL-High	WNL	CND	Central	98	96	68	80	11.8	5.00	8.75	WNL	WNL
**20**	23	M	WNL	WNL	ABNL-Low	ABNL-Inc	WNL	WNL	WNL	ND	ND	Peripheral	96	78	18	52	5.5	6.25	8.75	WNL	WNL
**22**	37	F	DNT	DNT	WNL	WNL	WNL	WNL	WNL	WNL	WNL	Normal	98	99	78	82	DNT	10.00	13.75	WNL	WNL
**25**	34	F	DNT	DNT	WNL	WNL	WNL	DNT	DNT	ND	WNL	Unknown	0	73	0	46	DNT	8.75	11.25	DNT	WNL
**26**	53	M	WNL	WNL	WNL	WNL	WNL	WNL	WNL	WNL	WNL	Normal	95	90	67	76	9.8	6.25	6.25	WNL	WNL
**27**	40	F	WNL	DNT	ABNL-High	WNL	WNL	ABNL-High	WNL	WNL	WNL	Central	97	86	34	69	11.0	6.25	10.00	WNL	WNL
**28**	61	F	WNL	DNT	WNL	WNL	WNL	DNT	DNT	WNL	CND	Unknown	79	87	38	61	12.4	17.50	15.00	WNL	DNT
**29**	58	F	WNL	DNT	WNL	ABNL-Inc	WNL	WNL	WNL	WNL	WNL	Unknown	95	90	57	76	9.6	11.25	15.00	ABNL	WNL
**30**	34	F	WNL	DNT	WNL	WNL	WNL	DNT	DNT	WNL	WNL	Normal	96	89	77	81	DNT	10.00	6.25	WNL	WNL
**31**	44	F	WNL	WNL	WNL	WNL	WNL	WNL	WNL	WNL	WNL	Normal	94	93	67	79	DNT	16.25	13.75	WNL	WNL
**32**	29	F	WNL	WNL	WNL	WNL	WNL	WNL	WNL	WNL	WNL	Normal	100	96	72	82	DNT	11.25	10.00	WNL	WNL
**33**	50	F	WNL	DNT	WNL	ABNL-Inc	WNL	WNL	DNT	ABNL-Low (1/4)	WNL	Unknown	95	97	76	78	DNT	DNT	DNT	DNT	DNT

Abbreviations: CM# = Chiari Malformation Patient Number; Age presented in years; cVEMP = Cervical Vestibular Evoked Myogenic Potential; oVEMP = Ocular Vestibular Evoked Myogenic Potential; VOR = Vestibular Ocular Reflex; Sym = Symmetry; Sup = Suppression; SOM = Somatosensory; VIS = Visual; VEST = Vestibular; SOT-Comp =Sensory Organization Test Comprehensive Score; A-P LOS = Anterior-Posterior Limits of Stability; PTA = Pure-tone Average; LE = Left Ear; RE = Right Ear; ABR = Auditory Brainstem Response; SPCH = Speech Discrimination Result; DNT=Did Not Test; ND = Not Done; CND = Could Not Determine; WNL = Within Normal Limits.; M = Male; F = Female; Amp = Amplitude; Lat = Latency; ABNL = Abnormal; Dec = Decreased; Inc = Increased. Alternate row shading is added for reading clarity.

**Table 4:** Summary of Vestibular Abnormalities.

Assessment	Total Tested	Number Abnormal	% Abnormal
**Cervical VEMP**	**19**	**2**	**10.5**
**Ocular VEMP**	**10**	**0**	**0**
**Total Rotational Chair**	**24**	**8**	**33.3**
Sinusoidal Harmonic Acceleration	24	8	33.3
Low Velocity Step	19	2	10.5
High Velocity Step	19	0	0
VOR Angular Suppression	16	1	6.3
Visual-Vestibular Enhancement	13	2	15.4
**Total SOT**	**23**	**11**	**39.1**
SOT Somatosensory	23	6	26.1
SOT Visual	23	3	13.0
SOT Vestibular	23	7	30.4
SOT Composite	23	6	26.1
Anterior-Posterior LOS	11	1	9.1
SOT Composite	23	6	26.1
Anterior-Posterior LOS	11	1	9.1

## Data Availability

Data sharing not available for this dataset secondary to informed consent restrictions precluding an appropriate level study replicability.
